# Modulation of microglial/macrophage activation by macrophage inhibitory factor (TKP) or tuftsin (TKPR) attenuates the disease course of experimental autoimmune encephalomyelitis

**DOI:** 10.1186/1471-2172-8-10

**Published:** 2007-07-16

**Authors:** Madhuri Bhasin, Muzhou Wu, Stella E Tsirka

**Affiliations:** 1 Genetics, Department of Pharmacological Sciences, University Medical Center at Stony Brook, Stony Brook, NY 11794-8651, USA; 2 Neuroscience, Department of Pharmacological Sciences, University Medical Center at Stony Brook, Stony Brook, NY 11794-8651, USA; 3 Molecular and Cellular Pharmacology, Department of Pharmacological Sciences, University Medical Center at Stony Brook, Stony Brook, NY 11794-8651, USA

## Abstract

**Background:**

Myelin Oligodendrocyte Glycoprotein (MOG)-induced experimental autoimmune encephalomyelitis (EAE) is the most commonly used mouse model for multiple sclerosis (MS). During the of progression of EAE, microglia, the immunocompetent cells of the brain, become activated and accumulate around demyelinated lesions. Microglial activation is mediated by the extracellular protease tissue Plasminogen Activator (tPA), and mice lacking tPA display altered EAE progression. In this study, we have used pharmacological inhibitors and stimulators of microglial/macrophage activation to examine the temporal requirement for microglial activation in EAE progression and to determine whether such approaches might potentially be of therapeutic value.

**Results:**

Intervention using the tripeptide macrophage/microglia inhibitory factor MIF (TKP) and the tetrapeptide macrophage/microglial stimulator tuftsin (TKPR) attenuated EAE symptoms and revealed that the timing of macrophage/microglial activation is critical for the clinical outcome of EAE. We show that the disease progression can potentially be manipulated favorably at early stages by altering the timing of microglial activation, which in turn alters the systemic immune response to favor upregulation of T helper cell 2 genes that promote recovery from EAE.

**Conclusion:**

Preventative and therapeutic modulation of macrophage/microglial activity significantly alters the outcome of EAE at symptomatic stages. Specific molecular targets have been identified that represent potential avenues of exploration for the treatment and prevention of MS.

## Background

Multiple sclerosis (MS) is a central nervous system (CNS) autoimmune disease with symptoms that include neurological impairment and motor deficits. MS results from immune attack on myelin, which leads to axonal and neuronal degeneration. A commonly used MS animal model is experimental autoimmune encephalomyelitis (EAE). EAE does not occur spontaneously, but does mimic some of the pathological and histological hallmarks of MS. During EAE, T cells recognizing components of myelin become activated, migrate to the CNS and cause autoimmune inflammation [1], which results in CNS infiltration of CD4+, CD8+ T cells and B cells. The inflammatory process includes secretion of proinflammatory T helper1 (Th1) cytokines [2] and an imbalance between Th1, T helper2 (Th2) and regulatory T cells [3,4]. When the ratio of Th1 to Th2 cells favors a predominantly Th2 profile, the proinflammatory properties of Th1 cytokines are countered and the severity of autoimmune diseases is alleviated [5,6]. Administration of soluble Tim-2, which is expressed preferentially by differentiated Th2 cells, to mice prior to EAE induction, results in Th2 cytokine overproduction and EAE symptom amelioration [7]. Recently another subclass of Th cells, the Th17, have been shown to be involved in the modulation of EAE symptoms, acting primarily through the cytokine IL-17 [8].

Microglia, which are macrophage-related cells resident in the CNS, play crucial roles in CNS injury [9-11]. Tools to study microglial involvement include pharmacologic small molecules that stimulate or block their activation. Tuftsin (threonine-lysine-proline-arginine, TKPR) promotes phagocytic activity for cells of monocytic origin that express tuftsin receptors, such as neutrophils, macrophages and microglia [12,13]. Tuftsin-positive cells are recruited to sites of inflammation, and the avidity and specificity of tuftsin for its receptor are sufficiently strong to enable exploitation of tuftsin for imaging and therapeutic purposes [14-16].

MIF (the tripeptide threonine-lysine-proline, tuftsin fragment 1–3, TKP) [17] inhibits macrophage/microglial activation by an unknown mechanism. In retrograde retinal ganglion cell degeneration, MIF retards neuronal death, enhances axonal regeneration, and elicits morphological transformation of activated microglia into oval, less ramified shapes [18]. MIF is an effective inhibitor of microglial activation and neurodegeneration in the mouse hippocampus during episodes of excitotoxicity [19].

An endogenous factor that triggers microglial activation is the serine protease tPA, which converts plasminogen into plasmin. tPA activity increases ten-fold in MS lesions and MS patients' cerebrospinal fluid during the acute disease phase, but is not increased in chronic MS [20,21]. tPA mRNA and activity increase in mice over the course of EAE [22]. tPA's binding partner, annexin II [23] and the remainder of the plasminogen activation system [24] are also upregulated in MS lesions.

tPA-deficient (tPA^-/-^) mice display altered EAE course compared to wild-type mice [22]. The onset of symptoms is delayed, indicating a contribution of tPA to the disease process; however, the extent of disease eventually exceeds that observed in wild-type mice and lasts longer, revealing an additional role for tPA in the recovery process. tPA is critical for neuropathology in other model systems such as excitotoxicity: tPA^-/- ^mice are less susceptible to glutamate-induced neurodegeneration in the hippocampus [25]. Interestingly, early pharmacological blocking of AMPA/KA glutamate receptors results in EAE amelioration, indicating that glutamate-induced excitotoxicity contributes to EAE neurological malfunction [26,27]. Excitotoxicity is accompanied by microglial activation and further glutamate release. A component of the complex tPA role in EAE may include alterations in microglial dynamics, since tPA^-/- ^mice exhibit attenuated microglial activation [25]. Supporting this hypothesis, active MS and EAE lesions are characterized by activated microglia [28], which have been shown to promote neurodegeneration in systems that model ischemic episodes [29].

In this study, we examine the consequences of altering the timing of macrophage/microglial activation during the course of EAE, and show that manipulating this pathway at the onset of clinical symptoms improves outcome, potentially by promoting a bias towards a Th2 phenotype.

## Results

### Inhibition of microglial activation prior to EAE induction has only modest effects on EAE progression

Given that the role of both tPA and microglia in EAE appears to be complex in nature, i.e. both degenerative and protective mechanisms appear to operate, we elected to examine the consequences of both microglial activation and microglial inhibition, and to do so at different time points in the course of the disease.

**Figure 1:**
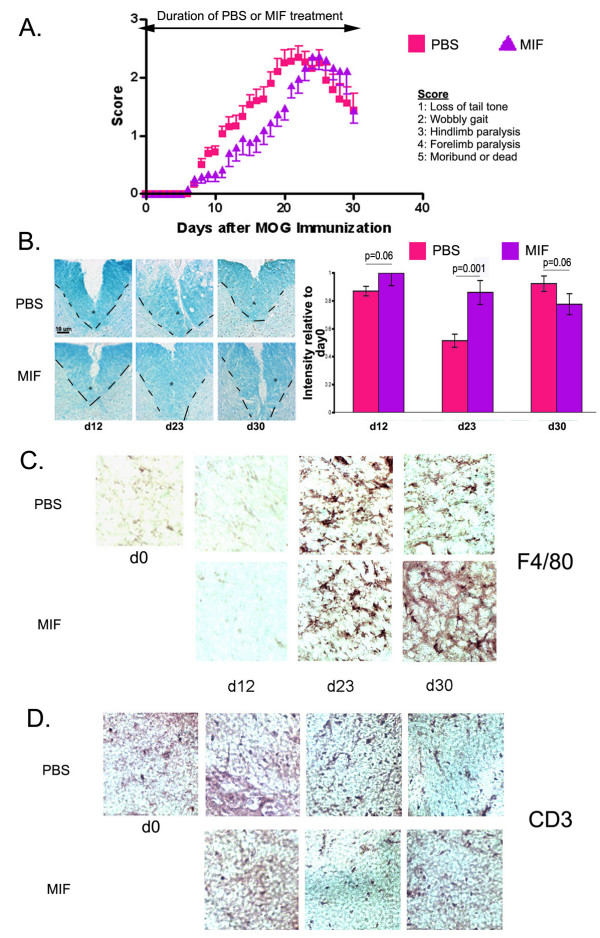
**Prophylactic MIF infusion does not alter onset, severity or recovery of EAE**. (A) Wt mice were infused with MIF for 30 days, starting one day prior to EAE induction. The mice were weighed and scored daily for symptoms using the severity scale outlined in *Methods*. Daily scores were averaged. PBS n = 18; MIF d-1 n = 12. Wilcoxon test showed no statistically significant differences in onset, severity or recovery, but the progression of the disease is more delayed. (B) Frozen spinal cord sections were stained with Luxol Fast Blue to show areas of myelination. The dashed line and the asterisk demarcate the ventral column of the spinal cord. The intensity of luxol fast blue staining within the ventral column was quantified using the NIH Image freeware and was normalized to day 0 staining. Background staining was subtracted. (C) Immunohistochemistry for activated macrophage/microglial cells using F4/80 in frozen spinal cord sections. (D) Immunohistochemistry for infiltrating T cells using an anti-CD3 antibody.

We began by assessing the outcome of prophylactically inhibiting microglial activation, i.e., administering an activation inhibitor the day before the first injection of MOG. For MOG-injected, PBS-treated (control) mice, onset of EAE symptoms occurred at day 7 (d7) and progressively worsened until d21, after which the mice began to recover (Fig. 1A). Prophylactic treatment with the macrophage/microglial activation inhibitor MIF resulted in a modest decrease in the rate of progression of the disease, although the day of onset, ultimate severity and rate of recovery were not dramatically different those observed for the control mice.

Loss of myelination was evaluated at different time-points. At d12, when the symptoms were still mild in nature, the PBS-infused mice exhibited normal levels of myelination (Fig. 1B). However, by d23, at which time the height of severity of symptoms was reached, extensive demyelination was apparent. Remyelination occured by d30, in agreement with the decline of clinical disease symptoms. In contrast, significant loss of myelination was not observed at any time point in the MIF-infused mice, consistent with the modestly improved clinical scores.

Macrophage/microglial activation was not detected in spinal cord sections from PBS- or MIF-treated mice at or before d12 (Fig. 1C). By day 23, however, many activated, amoeboid macrophage/microglia were observed in the PBS-treated mice, and, unexpectedly, significant activation was also seen in the MIF-treated mice. By day 30, the macrophage/microglial activation had subsided in the PBS-treated mice, in agreement with our previous reports (Lu et al., 2002), and similar findings were observed for the MIF-treated mice. In contrast, a slight reduction of infiltration of T cells into the spinal cord may have occurred for the MIF-treated animals as assessed by the T cell marker CD3 (Fig 1D).

Taken together, a moderate improvement in clinical outcome and demyelination was observed when the mice were treated with MIF before MOG immunization, despite the absence of complete inhibition of macrophage/microglial activation. Both morphological activation and migration to sites of injury are required for microglia to carry out neurotoxic actions [30]; in this instance, the clinical benefit observed may have ensued from interfering more with the migration than with the morphological activation.

### Inhibition of microglial activation at EAE onset markedly decreases EAE progression

**Figure 2:**
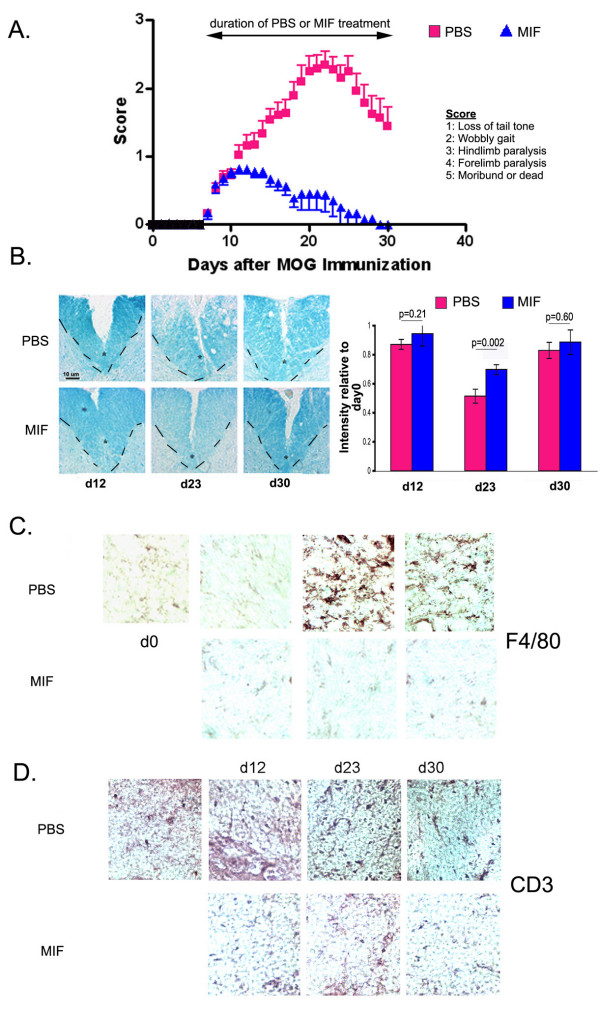
**Therapeutic MIF infusion severely abrogates the course of EAE**. (A) No statistically significant difference was observed in EAE onset between wt PBS and wt MIFd7 mice. However, the symptom severity was dramatically decreased (p = 0.0005) in wt MIFd7 compared to wt PBS; recovery was complete (p = 0.0005). wt PBS n = 18; wt MIF d7 n = 11. (B) Luxol Fast Blue staining shows very little demyelination at day 23 in wt MIFd7 mice. The dashed line and the asterisk demarcate the ventral column of the spinal cord. The intensity of luxol fast blue staining within the ventral column was quantified using the NIH Image freeware and was normalized to day 0 staining. Background staining was subtracted. (C) Immunohistochemistry for reactive macrophages/microglia using antibody against F4/80. (D) Immunohistochemistry for CD3+ T cells.

Given the modest improvement observed in Fig. 1 after early intervention with MIF, an unexpectedly positive outcome was obtained upon therapeutic use of MIF (initiating treatment at d7 after MOG injection), which elicited a dramatic abrogation of EAE symptoms and complete recovery by d27 (Fig. 2A). Histological decreases in myelination (Fig. 2B) were not observed for the MIF-treated animals, and, in contrast to what was observed for the prophylactic treatment using MIF, macrophage/microglial activation was also not observed (Fig. 2C), and there was a more apparent reduction of infiltration of T cells into the spinal cord (Fig. 2D). Multiple possibilities could account for this difference in outcome, as presented in the Discussion section.

### Stimulation of macrophage/microglial activation before the onset of EAE in wild-type mice promotes low level EAE and earlier recovery from symptoms

The pharmacological manipulation of macrophage/microglial status with MIF in the wt mice suggested that, as in other paradigms [18], macrophage/microglia can affect disease outcome and progress. Therefore, we sought to determine the effect on EAE of prematurely activating macrophage/microglia in wild-type mice.

**Figure 3:**
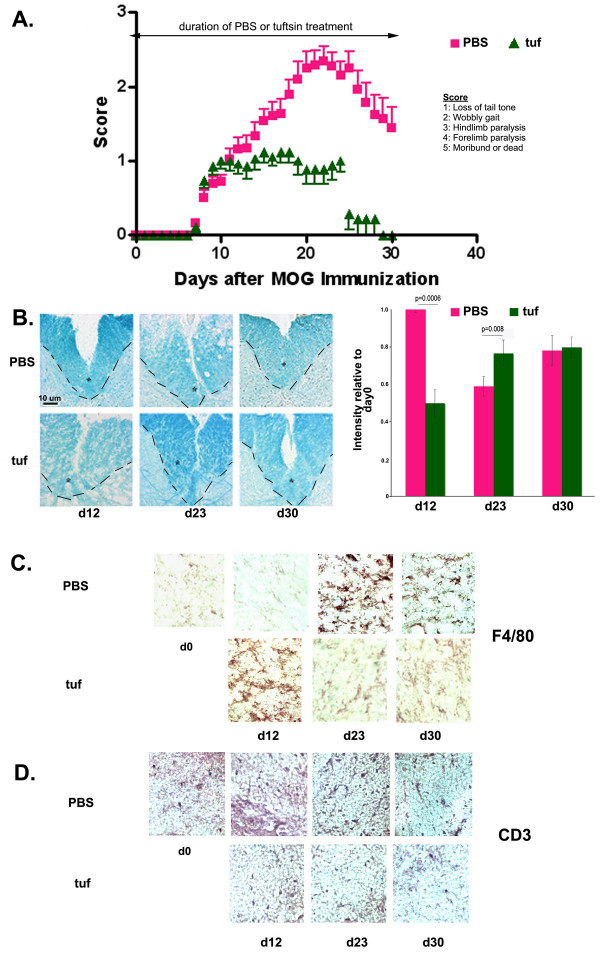
**Prophylactic administration of Tuftsin in wild-type mice results in a dampened disease course**. (A) Disease onset in wt tufd-1 mice does not differ from wt PBS mice. Severity is dramatically decreased (p = 0.008) and recovery is complete (p = 0.016). wt PBS n= 18, wt tufd-1 n = 13. (B) Luxol fast blue histological stain reveals levels of myelination. The dashed line and the asterisk demarcate the ventral column of the spinal cord. The intensity of luxol fast blue staining within the ventral column was quantified using the NIH Image freeware and was normalized to day 0 staining. Background staining was subtracted. (C) Reactive macrophages/microglia are visible in the coronal sections of experimental mice at different timepoints. (D) Infiltrating T cells were detected by immunohistochemistry using an anti-CD3 antibody.

Infusion of tuftsin one day prior to induction of EAE (tuf) resulted in decreased symptom severity and earlier recovery, although the day of onset was unaffected (Fig. 3A). The symptoms plateaued at a low level (clinical score of 1) before complete recovery was observed around day 29.

Significant demyelination in tuf-treated mice was evident at d12 compared to the PBS-treated animals (Fig. 3B), but remyelination occurred faster and was already detectable at d23.

At day 12, while both PBS- and tuf-treated mice had similar disease scores, the wt tufd-1 animals displayed strong immunoreactivity for F4/80, the marker for microglial activation (Fig. 3C). At later timepoints however, despite the infusion of tuftsin, no macrophage/microglial activation was evident in wt tufd-1 compared to wt PBS mice. The lack of macrophage/microglial activation was accompanied by virtual absence of CD3+ cells in the lesioned spinal cord (Fig. 3D).

### Stimulation of macrophage/microglial activation at the onset of EAE in wild-type mice attenuates EAE symptoms

**Figure 4:**
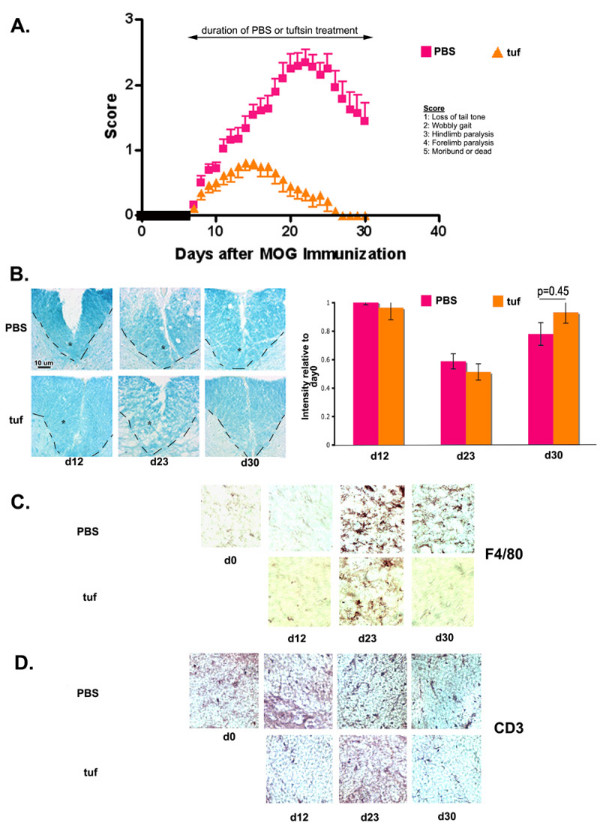
**Therapeutic use of Tuftsin in wt mice results in a severely abrogated course of disease**. (A) Disease onset is the same in wt tufd7 mice and wt PBS mice. Severity is strongly dampened (p = 0.004) and recovery is complete (p = 0.0002). wt PBS n= 18, wt tufd7 n = 13. (B) Levels of myelination are visualized using luxol fast blue. The dashed line and the asterisk demarcate the ventral column of the spinal cord. The intensity of luxol fast blue staining within the ventral column was quantified using the NIH Image freeware and was normalized to day 0 staining. Background staining was subtracted. (C) Macrophage/microglial activation is visualized with F4/80 staining. (D) Immunohistochemistry for infiltrating T cells using an anti-CD3 antibody.

Infusion of tuftsin in wild-type mice 7 days after MOG immunization (tuf) resulted a dramatic attenuation of the clinical symptoms (Fig. 4A). Minimal demyelination was observed in tuf-treated mice at d12 (Fig. 4B), but the demyelination increased by d23; however, by d30 the remyelination process became evident and extended to levels comparable to those in PBS-treated animals.

At day 12, tuf-treated mice showed little activated macrophage/microglial immunoreactivity (Fig. 4C). However by day 23, a limited degree of macrophage/microglial activation observed. Very limited infiltration of CD3+ cells was observed in the tuf-treated spinal cords at all time-points (Fig 4D).

### Early stimulation of macrophage/microglial activation in tPA-deficient mice accelerates the onset of EAE-like symptoms but prevents disease progression to severe levels

Since inhibiting macrophage/microglial activation in wild-type animals at the onset of EAE symptoms decreased the severity of disease, we next examined whether provoking macrophage/microglial activation in a setting where it is not maximal would exacerbate the progression of EAE. We used tPA-deficient animals, which display attenuated activation of microglia in comparison to wild-type animals in the context of excitotoxic and EAE-elicited injury [25,22]. Tuftsin was employed to activate macrophage/microglia in the animals prior to or during the course of EAE.

**Figure 5:**
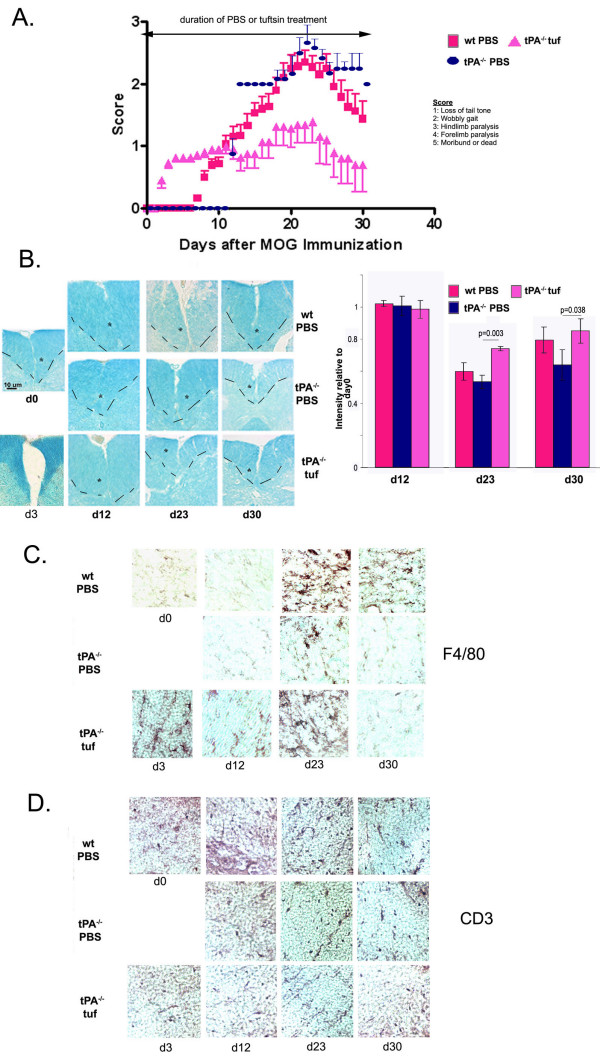
**Prophylactic tuftsin infusion at day -1 in tPA^-/- ^mice results in a dramatically early onset of EAE**. (A) Disease onset occurs at day 3 in tPA^-/- ^tufd-1 mice, much earlier than wt PBS (p = 0.03). Severity is dramatically decreased (p = 0.03) and recovery remained at a low level (p = 0.08). wt PBS n= 18, tPA^-/- ^tufd-1 n = 11. (B) Luxol Fast Blue staining to visualize levels of demyelination. The dashed line and the asterisk demarcate the ventral column of the spinal cord. The intensity of luxol fast blue staining within the ventral column was quantified using the NIH Image freeware and was normalized to day 0 staining. Background staining was subtracted. (C) F4/80 immunohistochemistry for activated macrophages/microglia. (D) Immunohistochemistry for infiltrating T cells using an anti-CD3 antibody.

Prophylactic administration of tuftsin to tPA^-/- ^mice resulted in early onset (d3) of EAE-like symptoms (Fig. 5A) in comparison to PBS-treated wt mice (onset at day 7) or tPA^-/- ^mice (onset at day 10, data not shown). Despite the early onset, however, a blunted disease progression was observed, i.e. the symptoms never achieved the degree of severity observed in PBS-treated mice.

No loss of myelination was observed at d12 (Fig. 5B). However, at the time-point with the most severe clinical score, day 23, the tuftsin-treated mice showed low levels of demyelination in the ventral column of the spinal cord, though much less so than that observed in sections prepared from wt mice treated with PBS. At day 30, there were lingering low levels of demyelination in the tPA^-/- ^tuftsin-treated mice.

Macrophage/microglial activation, albeit limited, was visible in tPA^-/- ^tuf-treated at d-1 mice as early as day 3 (Fig. 5C) and persisted for the duration of the period examined. However, the number of activated macrophages/microglia observed at d23 and d30, while higher than that observed for tPA^-/- ^mice treated with PBS, was not as high as that observed for wild-type. The number of infiltrating CD3+ cells was also diminished in all timepoints for the tPA^-/- ^prophylactically treated with tuftsin (Fig. 5D).

Although the symptoms observed at day 2 appeared EAE-like, it was possible that the tuftsin provoked an effect unlinked to the MOG injection. To confirm that the symptoms were genuinely due to early onset EAE, we infused tuftsin at d-1, and then injected complete Freund's adjuvant and pertussis toxin with substitution of PBS for the MOG. None of the mice exhibited any symptoms on the EAE clinical score scale (data not shown), indicating that the early onset of symptoms in the tPA^-/- ^tuf-treated at day-1 mice is dependent on the MOG immunization.

### Stimulation of macrophage/microglial activation in tPA-deficient mice as EAE symptoms start does not affect the course of EAE

**Figure 6:**
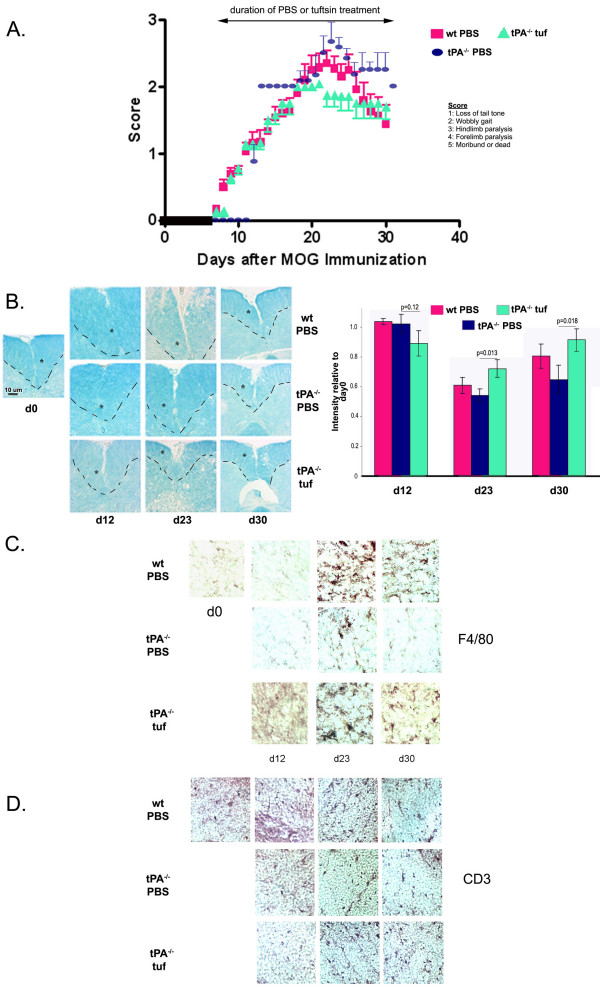
**Therapeutic administration of tuftsin in tPA^-/- ^mice does not alter onset, severity or recovery of EAE**. (A) Wilcoxon test showed no significant difference in EAE parameters (p > 0.05). wt PBS n = 18, tPA^-/- ^tufd7 n = 7. (B) Demyelination is visualized by luxol fast blue. The dashed line and the asterisk demarcate the ventral column of the spinal cord. The intensity of luxol fast blue staining within the ventral column was quantified using the NIH Image freeware and was normalized to day 0 staining. Background staining was subtracted. (C) F4/80^+^-reactive macrophages/microglia detected by immunohistochemistry. (D) Immunohistochemistry for T cells using an anti-CD3 antibody.

Therapeutic use (day 7) of tuftsin in tPA^-/- ^(tPA^-/- ^tuf) mice resulted in a disease pattern that was statistically similar to the wild-type disease profile, with a similar day of onset, day of peak severity, and course of recovery (Fig. 6A). Peak severity was slightly lower in the tuftsin-treated animals, but the difference was not statistically significant.

As we have previously reported [22], no loss of myelination levels is observed for MOG-injected wt or tPA^-/- ^mice at the d12 timepoint (Fig. 6B). Demyelination is observed by d23 in wt mice ([22] and Fig. 1B), but is delayed in tPA^-/- ^mice with luxol fast blue (LFB) persistent loss being observed at d30, by which time wt mice have remyelinated. In contrast, readily detectable areas of demyelination were observed in the tPA^-/- ^tuf mice at d12. At day 30, when tPA^-/- ^PBS mice still exhibit loss of myelination, remyelination was detected in the tPA^-/- ^tuf, mice similar to wt PBS mice.

Levels of macrophage/microglial activation in tPA^-/- ^tuf mice were higher at day 12 than that observed for wild-type mice treated with PBS (Fig. 6C). By day 23, both the wt PBS-treated mice and the tPA^-/- ^tuf-treated mice displayed robust and comparable levels of activated macrophage/microglia. In contrast, macrophage/microglia in the tPA^-/- ^mice treated with PBS exhibited only attenuated activation. By day 30, the macrophage/microglia in wt PBS-treated mice had begun to revert to a resting morphology, but remained activated in the tPA^-/- ^tuf-treated mice. The numbers of CD3+ cells were comparable between tPA^-/- ^PBS and tPA^-/- ^tuf animals, but were attenuated in numbers in comparison to those found in wt PBS mice (Fig 6D).

### MIF and tuftsin decrease the activity of Angiotensin Converting Enzyme

Angiotensin Converting Enzyme or ACE catalyzes the conversion of Angiotensin (Ang) I to Ang II. Ang II has been implicated in inflammation [31] and the upregulation of the expression of adhesion molecules thus influencing the permeability of blood-brain barrier [32]. It has been shown that administration of the ACE inhibitor captopril (containing the dipeptide KP) has a beneficial effect on EAE in Lewis rats [32]. Furthermore, tuftsin has been shown to decrease functions of angiotensin II by 20% [33]. Since MIF (TKP) and tuftsin (TKPR) differ by only one amino acid at the tuftsin C-terminus, we sought to determine whether MIF or tuftsin would function as an ACE inhibitor. When ACE activity was quantified in the presence of MIF or tuftsin, we found that MIF decreased ACE activity by 33% and tuftsin inhibited ACE activity by 24% (data not shown). This result indicates that the two compounds could function as ACE inhibitors to decrease the systemic immune reaction, but does not address their differential effect on the course of EAE.

### A Th2 shift in the immune response

**Figure 7:**
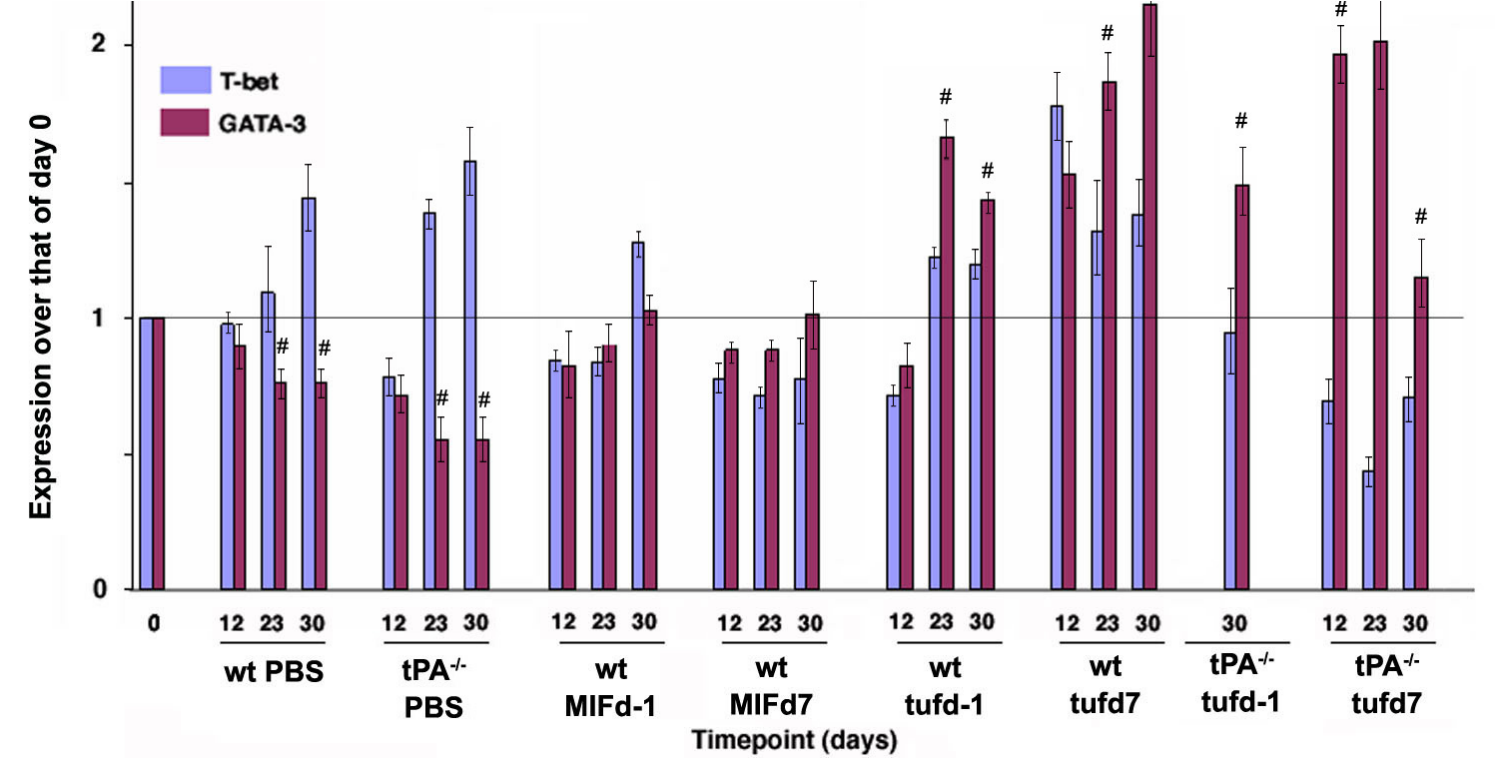
**Quantification of T-bet (Th1 transcription factor) and GATA-3 (Th2 transcription factor) expression**. Real time RT-PCR was performed on RNA from spinal cord homogenates extracted after the different treatments with MIF and tuftsin at various timepoints. Expression levels obtained for T-bet and GATA-3 were normalized against actin levels at the same timepoint in the same sample. **#**, denotes significant differences between the two (p < 0.05) groups for the specific treatment and timepoint.

Differential expression of the transcription factors T-bet and GATA-3 were used to analyze the Th1/Th2 balance in EAE mice in all conditions, since T-bet controls the transcription of Th1 markers and GATA-3 drives transcription of Th2 markers. PBS-treated, MOG-injected wt and tPA^-/- ^mice were characterized by a strong Th1 response, indicated by increasing levels of T-bet expression, and lowered levels of GATA-3. This result is in agreement with reports indicating that EAE is primarily a Th1-mediated disease [34]. However, both MIF and tuftsin administration into MOG-injected wt mice resulted in a shift towards increased GATA-3 expression, indicating a switch to a Th2 response. Similarly, the infusion of tuftsin in tPA^-/- ^mice resulted in a strong Th2 response (Fig 7). These results were further corroborated by measuring the expression levels of Th1- (TNFalpha) and Th2- (IL10) specific cytokines by RT-PCR and ELISA in spinal cord extracts (data not shown).

## Discussion and conclusion

The data presented n the current study indicate that administration of macrophage/microglial modulators at different points during the course of EAE can dramatically affect the outcome of the disease. We used 4 different modalities for modifying macrophage/microglial activation in MOG-injected wild-type mice, of which three resulted in improved EAE clinical scores. MIF treatment as EAE symptoms start, or tuftsin treatment either before or at the time of symptom onset were effective, whereas pre-treatment with MIF was of limited benefit. The complexity of the disease does not readily allow us to offer a direct explanation of the molecular events that underlie the differences in disease outcome with the various modalities of manipulations of microglial activation. One correlation that connects the three successful modalities is the observation of a balanced T cell response with a predominance of a Th2 fate for the activated T cells (Fig. 7). Another factor that could come into play is the requirement for macrophages/microglia to migrate to local sites of injury. The MIF macrophage/microglial inhibitor may block migration more effectively than morphological activation (Fig. 1), which could still confer a (modestly) beneficial outcome, and accelerated activation by tuftsin might lead to in situ morphological activation of the macrophages/microglia than the usual chemotactic migration and proliferation followed by local morphological activation. This model would provide an explanation for why fewer activated macrophages/microglia are observed after tuftsin treatment in the areas of interest. Additional possibilities are discussed below. Regardless, however, the benefit of the intervention is clear.

In our previous work, we found that mice deficient in tPA exhibit an altered progression of EAE symptoms that included the timing of clinical disease onset and the recovery from disease [22]. As these mice have attenuated microglial activation, we explored the question here of whether it was this deficiency that was driving the altered EAE response by systemically delivering modulators of macrophage/microglial activation and evaluating their effect on the progression of EAE. Although using systemic delivery is most relevant clinically, one concern about it is whether the administered compounds reached the CNS in therapeutic levels. Our data indicate that the compounds delivered affected the status of cells in the CNS. Furthermore, when administering MIF/TUF prior to the induction of EAE, we observed in some cases dramatic effects very early on (for example in the case of tuftsin delivery at day-1 in tPA-deficient animals), ostensibly before efficient BBB breakdown.

Many treatments for MS have focused on controlling blood-brain barrier permeability; inflammation is linked to the opening of the blood-brain barrier, since it allows infiltration of inflammatory cells into the CNS. Activated microglia release cytokines and chemokines that draw and activate leukocytes via the compromised blood-brain barrier [35]. Numerous agents have been used that are either anti-inflammatory themselves, or increase the secretion of anti-inflammatory cytokines and decrease that of pro-inflammatory cytokines [36].

The activation process of the endogenous microglia, which converts them from resting ramified cells to immunocompetent inflammatory ones, is associated with antigen presentation, myelin and tissue breakdown, production of reactive oxygen and nitrogen species and pro-inflammatory cytokines [37,38]. In the absence of activation, such as in the presence of an activation inhibitor like MIF, microglia are unable to mediate these effects [18]. The inhibition of microglial activation could thus be responsible for the diminished demyelination observed in the MIF-infused mice.

It is possible, on the other hand, that demyelination does occur in the MIF-infused mice, albeit to a lower degree. However, any myelin debris that is present might fail to be cleared due to the absence of activated macrophages/microglia. Consistent with this possibility, Luxol Fast Blue would stain degradation products of myelin lipoproteins that have not been phagocytosed by the inactivated macrophages/microglia, thus explaining the increased LFB staining despite the parallel symptomatology of MIF-infused and PBS-infused wt mice. Therapeutic (day 7) administration of MIF severely abrogated EAE symptoms. Therefore, the timing of macrophage/microglial inhibition could therefore be crucial to the outcome of the disease. The absence of activated microglia at the onset of the disease appears to have a beneficial effect.

Prophylactic activation of macrophages/microglia in tPA-deficient mice, which normally exhibit attenuated microglial activation [22], resulted in early onset of symptoms at day 3 that persisted for the duration of the experiment. Shaked *et al*., have shown in a model of optic nerve crush injury that earlier onset of phagocytic activity and antigen presentation by microglia results in resistance to injury and neuronal survival [9]. It is possible that early activation of microglia could have ameliorated EAE in a similar manner, by recruiting and interacting with the adaptive immune response rather than worsening it, potentially inducing protective autoimmunity.

Studies on protective autoimmunity have found that systemic T-cell responses are triggered by injury to CNS axons. The absence of mature T cells in some mouse strains results in impaired CNS recovery [39]. Thus early activation of microglia may have ameliorated the disease by presenting antigen to T helper cells and subsequently coordinating the resulting adaptive immune response. As suggested by Shaked *et al*, the balance between protective autoimmunity and autoimmune disease may be determined by the timing and intensity of microglial activation and initial immune response [9]. The Th2, anti-inflammatory switch that is evident in tPA-deficient mice after use of tuftsin provides further support to the idea that tuftsin may promote protective autoimmunity.

The protection conferred by early microglial activation diminished when the activation was delayed. Tuftsin administration at day 7 in tPA^-/- ^mice resulted in a very similar EAE course to that of PBS-infused mice. The therapeutic window for protection may be suboptimal when microglia are activated late, as demonstrated by Shaked *et al*. [9].

Our attempts to activate prematurely and in a sustained manner wild-type microglia did not always result in greater macrophage/microglial activation. In wt mice infused with tuftsin at d-1, greater levels of macrophage/microglial activation were observed at day 12 in comparison to wt PBS mice. However, when macrophage/microglial activation in wt PBS mice is detectable, at day 23 and day 30, the activation mediated by tuftsin seems to be reversed and fewer activated macrophages/microglia are observed (Fig 3C). This result suggests that there may be a control mechanism that does not allow sustained superactivation of macrophages/microglia. Despite the increased reaction however, the EAE symptoms are dampened. It is possible that macrophage/microglial activation during the induction phase of EAE once again acts to protect (precondition) against the disease. Once macrophage/microglial activation is induced normally by the disease process, tuftsin is no longer effective, potentially due to inability to superactivate the cells. In that case, the therapeutic window has already been exploited in the early stages.

Tuftsin infusion in wild-type mice at the onset of disease did not result in exaggerated macrophage/microglial activation at any timepoint (Fig 4C). Despite this result however, the disease course was severely abrogated. It is possible that premature activation of macrophage/microglial cells resulted in a paradoxical dampening of activation and reduced EAE intensity because of loss of antigen presentation, myelin and tissue breakdown, reactive oxygen species and pro-inflammatory cytokines, as mentioned above.

One explanation for the efficacy of MIF and tuftsin is that they both act eventually as anti-inflammatory agents, as they both can function as ACE inhibitors. Therefore in the context of EAE this function could result in reduction of inflammation and limited infiltration of immune cells from the systemic circulation [31]. Indeed, in a model of heart failure, it has been shown that ACE inhibitors reduce the ratio of Th1 cytokines to Th2 cytokines, which is indicative of a switch away from inflammation [40]. Furthermore, it has been shown that use of an ACE inhibitor, captopril, has a beneficial effect on EAE in Lewis rats [32]. On the other hand, while it is true that MIF and tuftsin may be acting as ACE inhibitors/anti-inflammatory agents, this is not universally true, as shown in Figs. 1 and 4, where although the two compounds are delivered, there is little change in EAE severity.

As the first line of defense in the CNS, microglia are critical determinants of the outcome of local injury. The timing and intensity of macrophage/microglial activation appear to be crucial to the course of the disease. Whether this effect is mediated by the interplay with T cells of the adaptive immune response and/or by modulation of inflammation, this study suggests that careful modulation of macrophage/microglial activation may be a viable therapeutic approach.

## Methods

All work with mice was approved by the Department of Laboratory Animal Resources at the State University of New York at Stony Brook. Mice were maintained under pathogen-free conditions at 21°C under a 12-hour light/dark cycle. Access to food and water was *ad libitum*.

### Induction of EAE

EAE was actively induced using myelin oligodendrocyte glycoprotein (MOG) 35–55 (MEVGWYRSPFSRVVHLYRNGK), as previously described [22,41]. MOG 35–55 was synthesized by Quality Controlled Biochemicals and purified using reverse-phase (C18) HPLC (QCB, Biosource, MA).

MOG35-55 (300 ug) was thoroughly homogenized with Freund's Adjuvant (Sigma, St. Louis, MO) containing 500 ug *mycobacterium tuberculosis *(Difco, Detroit, MI). This emulsion (200 ul) was injected into the flank of female mice (wild-type and tPA^-/-^), aged 6–10 weeks (day 0) along with 500 ng of pertussis toxin (List Biological Laboratories, Campbell, CA), which was injected ip in a volume of 200 ul. Two days later (day 2), the pertussis toxin was injected for a second time. On day 7, a second MOG injection was given in the opposite flank.

### Evaluation of EAE symptoms

An experimenter blinded to treatment conditions and genotypes monitored behavioral symptoms and weighed the animals daily. Symptom severity was assessed on a scale of 0 to 5 with intermediate scores being denoted by graduations of 0.5. The scale is as follows: 0, no symptoms; 1, loss of tail tone; 2, wobbly gait; 3, hindlimb paralysis; 4, forelimb paralysis; 5, moribund or dead [42].

### Time-controlled drug delivery

Alzet miniosmotic pumps (Durect, Cupertino, CA) were used to ensure time-controlled compound delivery. 14-day pumps (rate of infusion 0.5 ul/hr, 200 ul total volume) were filled with either PBS, 500 uM MIF (Sigma, St. Louis, MO) or 500 uM tuftsin (American Peptide Company, Sunnyvale, CA) and incubated overnight at 37°C, according to manufacturer's instructions.

Adult wild-type and tPA^-/- ^female mice (6–10 weeks old) were deeply anesthetized using i.p. atropine (0.6 mg/kg body weight) and 2.5% avertin (0.02 ml/g body weight). Pumps were implanted subcutaneously in the back of the animal for 14 days. Pumps were replaced at d14 with fresh 14-day pumps and were maintained for the duration of the experiment. The pumps containing PBS were implanted at the same time as the respective MIF and tuftsin pumps.

In early control experiments we confirmed that placement of a PBS pump either prior to (day -1) or after (day 7) EAE induction did not alter the course of disease. Therefore, all data for wild-type mice or tPA^-/- ^mice infused with PBS were pooled together from 4 independent experiments. Importantly, wt- or tPA^-/-^-PBS mice were used in every single experiment and ran as controls side-by-side with the experimental groups.

### Immunohistochemistry and histological stains

Spinal cords were harvested from mice at various timepoints over the course of the disease, and were fixed in 4% paraformaldehyde and 20% sucrose in PBS. The spinal cords were divided in three equal sections. The sections of each spinal cord were embedded in Tissue-Tek (Miles, Elkhart, IN) optimal cutting temperature compound, frozen on dry ice, and stored at -80°C until use. Coronal sections were obtained using a cryostat (Leica, Nussloch, Germany) and mounted onto slides (Superfrost Plus, Fisher Scientific), such that all three initial sections were represented. Slides were stored at -80°C until use.

After inhibiting endogenous peroxidase activity using 0.3% hydrogen peroxide, sections were blocked with serum overnight. F4/80, an antibody revealing macrophages/microglia, at 1:100 (Serotec, Raleigh, NC), was added to the sections for 1 hr at room temperature [43]. The sections were incubated with secondary antibody (Vector Labs) for 1 hour at room temperature, the ABC reagent (Vector Laboratories, Burlingame, CA) was added and diaminobenzidine was applied for visualization of the avidin-biotin complex [29]. Slides were then successively dehydrated, dipped in xylene and then coverslipped using Permount (Fisher Scientific, Pittsburgh, PA). In addition to F4/80 other markers were also used to visualizing the status of microglial activation, such as Isolectin B4, Iba1, and 5-D-4 (data not shown).

To evaluate the levels of myelination of individual sections, slides were dehydrated and incubated overnight at 56°C in 0.1% Luxol Fast Blue (Sigma, St. Louis, MO) in 95% ethanol and glacial acetic acid. The slides were then rinsed in 95% ethanol and distilled water and differentiated successively in 0.1% lithium carbonate and 70% ethanol. After dehydration and xylene treatment, the slides were coverslipped using Permount. The intensity of LFB labeling was quantified on multiple sections in each animal and was averaged and plotted.

### Angiotensin Converting Enzyme (ACE) assay

For the standard curve, varying volumes of 0.1 units/ml of ACE were incubated with 200 ul of 6.25 mM hyppuric acid (substrate) in 125 mM borate buffer. To test for inhibition of ACE activity, varying volumes of 0.5 mM MIF or Tuftsin were preincubated for 1 hour at room temperature before adding 200 ul of substrate. All samples were incubated with substrate for 90 minutes at 37°C in glass test tubes. The reaction was stopped by addition of 250 ul of 20 mM EDTA in borate buffer. 2 ml of borate buffer and 1.5 ml of 160 mM cyanuric chloride (resuspended in spectrophotometric grade Dioxane) were added. Tubes were centrifuged for 10 minutes at 6000×g. Absorbance of supernatants was read in a spectrophotometer at 405 nm against distilled water [44].

### T-bet and Gata-3 quantitative RT-PCR

RNA was extracted from spinal cord homogenates using Trizol (Invitrogen, CA). cDNA was synthesized using SuperScript™ II Reverse Transcriptase (Invitrogen, CA) as per the recommended protocol. Two μl of the diluted cDNA was used in a 20-μl realtime PCR reaction volume containing 3 mM MgCl_2_, 0.5 μM of each primer, and all other components as recommended by LightCycler^® ^FastStart DNA Master SYBR Green I kit (Roche Applied Science). The reactions were performed on LightCycler^® ^instrument (Roche Applied Science).

The primers used for PCR were:

T-bet (forward): GCCAGGGAACCGCTTATATG

T-bet (reverse): TCCCCCAAGCAGTTGACAGT

GATA3 (forward): CTGACTATGAAGAAAGAAGGCATCCAG

GATA3 (reverse): AAGTAGAAGGGGTCGGAGGAACTCT

β-Actin (forward): GGCCACTGCCGCATCCTCTT

β-Actin (reverse): AGAGCCTCAGGGCATCGGAAC

The PCR program for T-bet was 95°C for 10 min, then 40 cycles at 95°C (10 s), 62°C (5 s), and 72°C (20 s), followed by the standard melting curve. The PCR program for GATA3 was 95°C for 10 min, then 40 cycles at 95°C (10 s), 58°C (5 s), and 72°C (20 s), followed by the standard melting curve. The PCR program for β-Actin was 95°C for 10 min, then 45 cycles at 95°C (10 s), 65°C (5 s), and 72°C (5 s), followed by the standard melting curve.

### Statistics

All EAE graphs were analyzed using GraphPad Prism. The Wilcoxon test for nonparametric data was performed to compare differences between drug treatment and PBS EAE. Statistical analysis was done for three EAE parameters: onset, severity and recovery. Timepoints for analysis were chosen based on first and last day of each of the parameters for all mice in a set. Student's T-tests were performed to compare myelination levels between the control and experimental group at each timepoint. P values are listed on the relevant graphs.

## Abbreviations

tPA: Tissue plasminogen activator

EAE: experimental allergic encephalomyelitis

MOG: myelin oligodendrocyte glycoprotein

LFB: Luxol Fast Blue

PBS: phosphate buffered saline

ACE: angiotensin converting enzyme

MIF: macrophage/microglial inhibitory factor

## Authors' contributions

MB performed the EAE experiments (monitoring behavioral scores and weight of animals) and the ACE experiments, statistical analysis and wrote the first draft of the manuscript. MW assisted in the EAE experiments, did F4/80 and CD3 immunostainings, LFB stainings and quantifications and the real-time PCR experiments. SET designed experiments, analyzed results, finished the writing of the manuscript, oversaw the project. All authors read and approved the final manuscript.

## References

[B1] Mor F, Kantorowitz M, Cohen IR (1996). The dominant and the cryptic T cell repertoire to myelin basic protein in the Lewis rat. J Neurosci Res.

[B2] Behi ME, Dubucquoi S, Lefranc D, Zephir H, De Seze J, Vermersch P, Prin L (2005). New insights into cell responses involved in experimental autoimmune encephalomyelitis and multiple sclerosis. Immunol Lett.

[B3] Olivares-Villagómez D, Wensky AK, Wang Y, Lafaille JJ (2000). Repertoire Requirements of CD4+ T Cells That Prevent Spontaneous Autoimmune Encephalomyelitis. J Immunol.

[B4] Chen X, Oppenheim JJ, Winkler-Pickett RT, Ortaldo JR, Howard OM (2006). Glucocorticoid amplifies IL-2-dependent expansion of functional FoxP3(+)CD4(+)CD25(+) T regulatory cells in vivo and enhances their capacity to suppress EAE.. Eur J Immunol.

[B5] Yoles E, Hauben E, Palgi O, Agranov E, Gothilf A, Cohen A, Kuchroo V, Cohen IR, Weiner H, Schwartz M (2001). Protective autoimmunity is a physiological response to CNS trauma. J Neurosci.

[B6] Weaver A, Goncalves da Silva A, Nuttall RK, Edwards DR, Shapiro SD, Rivest S, Yong VW (2005). An elevated matrix metalloproteinase (MMP) in an animal model of multiple sclerosis is protective by affecting Th1/Th2 polarization. FASEB J.

[B7] Chakravarti S, Sabatos CA, Xiao S, Illes Z, Cha EK, Sobel RA, Zheng XX, Strom TB, Kuchroo VK (2005). Tim-2 regulates T helper type 2 responses and autoimmunity. J Exp Med.

[B8] Rohn TA, Jennings GT, Hernandez M, Grest P, Beck M, Zou Y, Kopf M, Bachmann MF (2006). Vaccination against IL-17 suppresses autoimmune arthritis and encephalomyelitis.. Eur J Immunol.

[B9] Shaked I, Porat Z, Gesner R, Kipnis J, Schwartz M (2004). Early activation of microglia as antigen-presenting cells correlates with T-cell mediated protection and repair of the injured central nervous system.. Journal of Neuroimmunology.

[B10] Drew PD, Storer PD, Xu J, Chavis JA (2005). Hormone regulations of microglial cell activation: relevance to multiple sclerosis. Brain Research Reviews.

[B11] Heppner FL, Greter M, Marino D, Falsig J, Raivich G, Hovelmeyer N, Waisman A, Rulicke T, Prinz M, Priller J, Becher B, Aguzzi A (2005). Experimental autoimmune encephalomyelitis repressed by microglial paralysis.. Nature Medicine.

[B12] Bump NJ, Najjar VA, Reichler J (1990). The characteristics of purified HL60 tuftsin receptors. Mol Cell Biochem.

[B13] Bump NJ, Lee J, Wleklik M, Reichler J, Najjar VA (1986). Isolation and subunit composition of tuftsin receptor. PNAS.

[B14] Paul C, Peers SH, Woodhouse LE, Thornback JR, Goodbody AE, Bolton C (2000). The detection and quantitation of inflammation in the central nervous system during experimental allergic encephalomyelitis using the radiopharmaceutical 99mTc-RP128. J Neurosci Meth.

[B15] Agrawal AK, Gupta CM (2000). Tuftsin-bearing liposomes in treatment of macrophage-based infections. Advanced Drug Delivery Reviews.

[B16] Fridkin M, Tsubery H, Tzehoval E, Vonsover A, Biondi L, Filira F, Rocchi R (2005). Tuftsin-AZT conjugate: potential macrophage targeting for AIDS therapy. J Pept Sci.

[B17] Auriault C, Joseph M, Tartar A, Capron A (1983). Characterization and synthesis of a  macrophage inhibitory peptide from the second constant domain of human immunoglobulin G.. FEBS Lett.

[B18] Thanos S, Mey J, Wild M (1993). Treatment of the adult retina with microglia-suppressing factors retards axotomy-induced neuronal degradation and enhances axonal regeneration in vivo and in vitro. JNeurosci.

[B19] Rogove AD, Tsirka SE (1998). Neurotoxic responses by microglia elicited by excitotoxic injury in the mouse hippocampus. Curr Biol.

[B20] Akenami FOT, Sirén V, Koskiniemi M, Siimes MA, Teräväinen H, Vaheri A (1996). Cerebrospinal fluid activity of tissue plasminogen activator in patients with neurological diseases. J Clin Pathol.

[B21] Virtanen JO, Zabriskie JB, Siren V, Friedman JE, Lyons MJ, Edgar M, Vaheri A, Koskiniemi M (2005). Co-localization of human nerpes virus 6 and tissue plasminogen activator in multiple sclerosis brain tissue.. Medical Science Monitor.

[B22] Lu W, Bhasin M, Tsirka SE (2002). Involvement of tissue plasminogen activator in both onset and effector phases of experimental allergic encephalomyelitis. J Neurosci.

[B23] Siao CJ, Tsirka SE (2002). Tissue plasminogen activator mediates microglial activation via its finger domain through annexin II. J Neurosci.

[B24] Teesalu T, Hinkkanen A, Vaheri A (2001). Coordinated induction of extracellular proteolysis systems during experimental autoimmune encephalomyelitis in mice. Am J Pathol.

[B25] Tsirka SE, Gualandris A, Amaral DG, Strickland S (1995). Excitotoxin induced neuronal degeneration and seizure are mediated by tissue-type plasminogen activator. Nature.

[B26] Pitt D, Werner P, Raine CS (2000). Glutamate excitotoxicity in a model of multiple sclerosis. Nature Med.

[B27] Smith T, Groom A, Zhu B, Turski L (2000). Autoimmune encephalomyelitis ameliorated by AMPA antagonists. Nature Med.

[B28] Ferguson B, Matyszak MK, Esiri M, Perry VH (1997). Axonal damage in acute multiple sclerosis lesions. Brain.

[B29] Tsirka SE, Rogove AD, Bugge TH, Degen JL, Strickland S (1997). An Extracellular Proteolytic Cascade Promotes Neuronal Degeneration in the Mouse Hippocampus. J Neurosci.

[B30] Ullrich O, Diestel A, Eyüpoglu IY, Nitsch R (2001). Regulation of microglial expression of integrins by poly(ADP-ribose) polymerase-1. Nature Cell Biol.

[B31] Das UN (2005). Is angiotensin-II an endogenous pro-inflammatory molecule?. Medical Science Monitor.

[B32] Constantinescu CS, Ventura E, Hilliard B, Rostami A (1995). Effects of the angiotensin converting enzyme inhibitor captopirl on experimental autoimmune encephalomyelitis.. Immunopharmacology Immunotoxicology.

[B33] Siemion IZ, Kluczyk A (1999). Tuftsin: On the 30-year anniversary of Victor Najjar's discovery. Peptides.

[B34] Huitinga I, Damoiseaux JG, Dopp EA, Dijkstra CD (1993). Treatment with anti-CR3 antobodies ED7 and ED8 suppresses experimental allergic encephalomyelitis in Lewis rats. European Journal of Immunology.

[B35] Petitto JM, Huang Z, Lo J, Streit W (2003). IL-2 gene knockout affects T lumphocyte trafficking and the micorglial response to regenerating facial motor neurons. Journal of Neuroimmunology.

[B36] Stanislaus R, Gilg AG, Singh AK, Singh I (2005). N-acetyl-L-cysteine ameliorates the inflammatory disease process in experimental autoimmune encephalomyelitis in Lewis rats.. Journal of Autoimmune Diseases.

[B37] Banati RB, Raivich G (2004). Brain microglia and blood-derived macrophages: molecular profiles and functional roles in multiple sclerosis and animal models of autoimmune demyelinating disease.. Brain Research Reviews.

[B38] Hendriks JJA, Teunissen CE, de Vries HE, Dijkstra CD (2005). Macrophages and Neurodegeneration. Brain Reserach Reviews.

[B39] Kipnis J, Yoles E, Schori H, Hauben E, Shaked I, Schwartz M (2001). Neuronal survival after CNS insult is determined by a genetically encoded autoimmune response. Journal of Neuroscience.

[B40] Gage J, Fonarow G, Hamilton M, Widawski M, Martinez-Masa O, Vredevoe D (2004). Beta blocker and angiotensin-converting enzyme inhibitor therapy is associated with decreased Th1/Th2 cytokine ratios and inflammatory cytokine production in patients with chronic heart failure. Neuroimmunomodulation.

[B41] Bernard CC, Johns TG, Slavin A, Ichikawa M, Ewing C, Liu J, Bettadapura J (1997). Myelin oligodendrocyte glycoprotein: a novel candidate autoantigen in multiple sclerosis. J Mol Med.

[B42] Hjelmstrom P (1998). B-cell-deficient mice develop experimental allergic encephalomyelitis with demyelination after myelin oligodendrocyte glycoprotein sensitization.. J Immunol.

[B43] Wang J, Tsirka SE (2005). Tuftsin fragment 1-3 is beneficial when delivered after the induction of intracerebral hemorrhage. Stroke.

[B44] Pre J, Bladier D (1983). A rapid and sensitive spectrophotometric method for routine determination of serum angiotensin I converting enzyme activity.. IRCS Medical Science.

